# The effectiveness of probiotic supplementation as an adjunct to phototherapy in the management of neonatal hyperbilirubinemia: a systematic review and meta-analysis

**DOI:** 10.1007/s11845-026-04434-8

**Published:** 2026-05-12

**Authors:** Sibel Yücetürk, Ayşe Çuvadar, Nurten Özçalkap

**Affiliations:** 1 Faculty of Health Sciences, Department of Midwifery, Sinop University, Sinop, Türkiye; 2 Faculty of Health Sciences, Department of Midwifery, Karabuk University, Karabuk, Türkiye; 3 Faculty of Health Sciences, Department of Midwifery, Ağri Ibrahim Cecen University, Ağri, Türkiye

**Keywords:** Probiotic supplement, Newborn hyperbilirubinemia, Bilirubin level, Meta-analysis

## Abstract

**Aim:**

This meta-analysis aims to determine the effect of probiotic supplementation, administered together with phototherapy, on hyperbilirubinemia levels in the treatment of neonatal jaundice.

**Method:**

This study was carried out between July and October 2024 utilizing the PubMed, Web of Science, EBSCOhost, National Thesis Center of the Council of Higher Education (YÖK), and Google Scholar databases. The data collection and analysis processes were conducted by three independent researchers. Fourteen eligible studies were included. Study quality was assessed with the Joanna Briggs Institute (JBI) tool. Data were analyzed using Comprehensive Meta-Analysis (CMA) Version 2. Findings were synthesized through meta-analysis and narrative methods.

**Results:**

Given the results of the meta-analysis based on the findings reported by the studies, probiotics administered in conjunction with phototherapy had a significant effect on hyperbilirubinemia levels among newborns (SMD: -0.364, 95% CI: -0.700 to -0.028; Z = -2.121, *p* = 0.034). Meta-analysis findings showed significant heterogeneity among studies (I²=88.7; *p* < 0.001). This high level of heterogeneity substantially reduces confidence in the pooled estimate and suggests that the effect size may vary across different clinical settings.

**Conclusıon:**

This meta-analysis suggests probiotics may be a complementary treatment for neonatal hyperbilirubinemia. However, these findings should be interpreted with caution due to the high heterogeneity and variability among included studies. Due to differences in probiotic strains, dosage, and methodology among the studies, the generalizability of the results is limited. This research was not funded by any institution or organization, and there are no conflicts of interest among the authors.

## Introduction

Neonatal indirect hyperbilirubinemia, commonly referred to as newborn jaundice, is generally benign, and mild elevations in bilirubin levels in term infants often do not require treatment [1]. However, jaundice develops in more than 50% of newborns within the first few days after birth, and 60–80% of these cases represent a significant global health issue that remains largely unpreventable [2]. Approximately 1.1 million newborns are affected annually, with the majority residing in South Asia and sub-Saharan Africa [3]. In term infants, hyperbilirubinemia may negatively impact hearing, motor skills, and neurological development [4, 5]. Elevated bilirubin levels were also shown to impair renal and cardiac functions [5], in addition to contributing to oxidative stress and DNA damage [6]. However, mild elevations in bilirubin were reported to have antioxidant effects at the cellular level and offer a potential protective role [6]. On the other hand, excessive accumulation of bilirubin in the brain can cause neurotoxicity, resulting in a severe condition known as kernicterus (bilirubin encephalopathy). Even though kernicterus can be life-threatening in the acute phase, it may also cause long-term irreversible neurological damage [7].

Main treatment modalities for neonatal jaundice include phototherapy, fluid supplementation, and exchange transfusion. Pharmacologic interventions may also involve agents such as phenobarbital, albumin, intravenous immunoglobulin (IVIG), metalloporphyrins, and probiotics [8]. Phototherapy is the main treatment for neonatal hyperbilirubinemia since it converts bilirubin into the water-soluble compound lumirubin and allow for excretion via urine and feces without the need for hepatic metabolism [9]. Probiotics, with their multifaceted and integrative effects, present a promising adjunctive approach for managing the complex condition of neonatal jaundice. Thanks to their low cost, ease of administration, and broad accessibility, probiotics offer a practical and potentially effective intervention that may be incorporated into future treatment strategies [10, 11]. Previous studies suggest that the relationship between gut microbiota and serum bilirubin levels is mediated through enterohepatic circulation [12]. Beneficial microorganisms in the gut facilitate the conversion of bilirubin to urobilinogen, which is then eliminated through feces. However, in neonates, the immaturity of the gastrointestinal system may limit the efficiency of this microbial process. This inefficiency can reduce the liver and biliary system’s ability to eliminate bilirubin from the body, thereby contributing to hyperbilirubinemia [13]. Several studies reported that when used in conjunction with phototherapy, probiotics may accelerate the reduction in bilirubin levels and shorten the duration of treatment [14–16].

There are many studies investigating the potential effects of probiotics in the treatment of neonatal hyperbilirubinemia. However, these earlier meta-analyses were limited by several methodological concerns, including the varying assessment of jaundice resolution times across different guidelines, inadequate reporting of randomization and blinding methods, small sample sizes, heterogeneity in the types of probiotics used, and high risk of bias, resulting in limited methodological consistency and reliability of findings. These limitations reduce the overall confidence in the pooled estimates and highlight the need for more rigorous and updated evidence. Therefore, updated and comprehensive studies that quantitatively assess the efficacy of probiotics used alongside phototherapy are needed [11, 17]. In this context, the present study provides an updated and systematic synthesis of current evidence, with a particular focus on methodological rigor and quantitative evaluation of treatment effects. This study aims to fill a gap in the literature by systematically analyzing existing evidence on the efficacy and safety of probiotic supplementation used in conjunction with phototherapy. Specifically, this study addresses the research question of whether probiotic supplementation, when used as an adjunct to phototherapy, significantly reduces bilirubin levels in neonates with hyperbilirubinemia. In this context, the effect of probiotic administration on the reduction of bilirubin levels in neonatal hyperbilirubinemia is evaluated. Overall, while probiotics may offer a potential supportive role, the findings should be interpreted with caution due to variability across studies. In this regard, the study contributes to the identification of potential supportive treatment approaches in the management of neonatal jaundice.

## Methods

This meta-analysis was structured in line with the PRISMA guidelines (Preferred Reporting Items for Systematic Reviews and Meta-Analyses) to ensure compliance with reporting standards [18]. To minimize the risk of bias and improve methodological reliability, the processes of literature search, study selection, and data extraction were conducted independently and in parallel by three researchers. To strengthen the reliability of these procedures, findings obtained at each stage were cross-checked and consensus was achieved through mutual verification. Moreover, the quality of the studies included in the meta-analysis was systematically assessed by the research team based on predefined criteria.

### Criteria for exclusion and inclusion

The studies in this meta-analysis were selected based on the PICOS framework (Population, Intervention, Comparison, Outcomes, Study Design):

- P (Population): Newborns requiring phototherapy.
- I (Intervention): Probiotic supplementation administered in conjunction with phototherapy.
- C (Comparison): Only newborns who received phototherapy.
- (Outcomes): Levels of hyperbilirubinemia.
- S (Study Design): Randomized controlled trials published in Turkish or English with an experimental design.

Publications that did not meet the methodological rigor, such as letters to the editor, quasi-experimental studies, qualitative research, single case reports, case-based narratives, and systematic or traditional reviews, were excluded from the analysis.

### Search strategy

The literature search was conducted between July and September 2024 using the PubMed, Web of Science, EBSCOhost, YÖK National Thesis Center, and Google Scholar databases. The search strategy incorporated MeSH-compliant terms, including: “Neonate” OR “NEONATAL” OR “Newborn” AND “Saccharomyces boulardii” OR “Bifidobacterium” OR “Lactobacillus” OR “probiotics” AND “neonatal hyperbilirubinemia” OR “neonatal jaundice.” All identified records were systematically transferred to the EndNote reference management software, and duplicates were removed.

### Selection of studies

A total of 9,785 records were identified through database searching. Following the removal of 750 duplicate records, 9,035 studies remained for the initial screening phase. During the title and abstract screening, several categories of ineligible studies were excluded based on predefined eligibility criteria. Specifically, 4,000 studies were removed for being out-of-scope or involving populations other than neonates; 100 records were excluded due to being published in languages other than English; 2,300 studies were eliminated because they involved non-probiotic interventions; 1,500 studies were excluded for investigating probiotic interventions that were not combined with phototherapy; and 1,102 studies were removed due to non-randomized or non-controlled study designs.

Following the review, the full texts of 33 studies that were considered to meet the predefined inclusion criteria (RCT, probiotic + phototherapy) were evaluated.

**Fig. 1:**
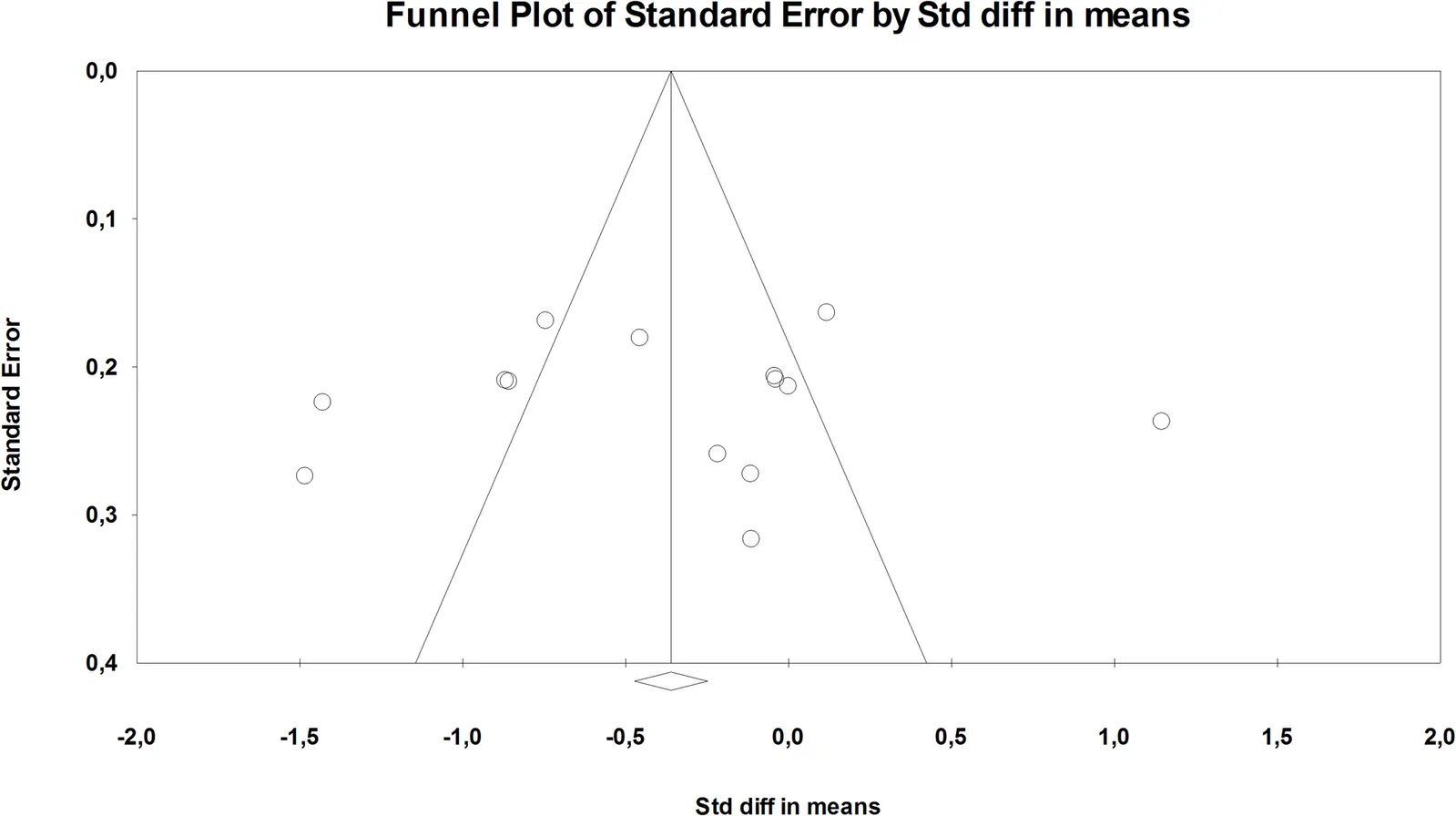
Selection of studies by following PRISMA flow diagram

At this stage, studies that only included probiotic intervention and did not apply phototherapy were excluded. Additionally, studies that did not clearly report bilirubin levels were not included in the evaluation. Although data were collected for one study, it was excluded from the analysis because the results had not yet been published and the completion date was specified as 2025. Furthermore, two studies that did not provide standard deviation values for bilirubin levels in the experimental and control groups were excluded because they did not provide suitable data for analysis. Another study was excluded because it did not meet the criteria, as it included six different probiotic intervention groups but did not involve phototherapy. As a result of this multi-layered exclusion process, a total of 14 studies were found to be suitable and included in the meta-analysis (Fig. 1).

Study selection and data extraction were performed by two independent authors, S.Y. and N.Ö., without blinding. Disagreements between authors were resolved through discussions involving a third reviewer, A.Ç.

### Data extraction

A data extraction form specifically developed by the researchers was utilized to systematically collect data within the scope of this study. Through this form, structured data were obtained regarding the author name and publication year, data collection period, country in which the study was conducted, measurement instruments used, sample size and characteristics, type of intervention, and main findings of the studies included in the meta-analysis (Table 1, Appendix Table 2). Additionally, the form includes the mean and standard deviation values of the study results.

**Table 1:** Characteristics and results of the included studies

Author/Year	Data Collection Period	Country/Continent	Sample Size	Intervention	Primary Outcomes
Abbas et al., 2023 [14]	August 2022 – November 2022	Indonesia	Intervention group: 20; Control group: 20	Experimental Group: Phototherapy + Probiotic supplementation (Lactobacillus acidophilus, Streptococcus thermophilus, Bifidobacterium longum; 5 drops/day for 7 days) Control Group: Phototherapy only	Total Bilirubin Levels (mg/dL): Intervention Group: 7.20 ± 2.80 Control Group: 7.48 ± 2.11
Afzal et al., 2021 [19]	April 2019 - September 2019	Islamabad	Intervention Group:30 Control Group:30	Intervention Group: Phototherapy + Bacillus clausii probiotic (2.5 mL, twice daily for 3 days) Control Group: Phototherapy only	Total Bilirubin Levels (mg/dL): Intervention Group:11.09 ± 2.87 mg/dL Control Group: 11.72 ± 2.96 mg/dL *p* > 0.05
Farhand et al., 2020 [20]	2020	Iran	Intervention Group:27 Control Group:27	Intervention Phototherapy + Lactoacidophilus probiotic Control Group: Phototherapy only	Total Bilirubin Levels (mg/dL): Intervention Group: -4.49 ± 2.62 mg/dL Control Group: -4.18 ± 2.76 mg/dL
Goodarz et al., 2022 [21]	2016	Iran	Intervention Group:65 Control Group:61	Intervention Group: Phototherapy + PediLact probiotic (Lactobacillus and Bifidobacterium strains) Control Group: Phototherapy only	Total Bilirubin Levels (mg/dL): Intervention Group: 16.57 ± 2.11 mg/dl Control Group: 17.39 ± 1.40 mg/dl
Habibi et al., 2021 [22]	April 2020 - February 2021	Iran	Intervention Group:44 Control Group:44	Intervention Group: Phototherapy + PediLact probiotic (5 drops/day during hospital stay) Control Group: Phototherapy + placebo (distilled water)	Total Bilirubin Levels (mg/dL): Intervention Group: 10.0 ± 2.06 mg/dL Control Group: 10.0 ± 1.49 mg/dL (*p* = 0.985.
Hu et al., 2023 [23]	February 2013 - March 2016	China	Intervention Group:55 Control Group:45	Intervention Group: Phototherapy + Saccharomyces boulardii (Florastor^®^, 250 mg/day for 15 days) Control Group: Phototherapy only	Total Bilirubin Levels (mg/dL): Intervention Group: 115.85 ± 26.67 mmol/L Control Group: 148.47 ± 48.48 mmol/L (*p* < 0.01).
Jenabi et al., 2022 [24]	October 2019 - June 2020	Iran	Intervention Group:75 Control Group:75	Intervention Group: Phototherapy + PediLact probiotic (10 drops/day, orally) Control Group: Phototherapy only	Serum Bilirubin Levels (SBL): Intervention Group: 7.2 ± 0.9 mg/dl. Kontrol: 7.8 ± 0.7 mg/dl
Liu et al., 2015 [25]	July 2012 - August 2014	China	Intervention Group:34 Control Group:34	Intervention Group: Phototherapy + Bifid Triple Viable Probiotic (2 g/day, administered in 3–4 doses) Control Group: Phototherapy only	Total Bilirubin Levels (mg/dL): Intervention Group: 79 ± 18 µmol/L Control Group: 108±21µmol/L (*p* = 0.001)
Mohammed et al., 2019 [26]	August 2017 - January 2018	Egypt	Intervention Group:50 Control Group:50	Intervention Group: Phototherapy + Probiotic (Lactobacillus acidophilus, Bifidobacterium lactis, Bifidobacterium bifidum, Lactobacillus rhamnosus) Control Group: Phototherapy only	Total Bilirubin Levels (mg/dL): Intervention Group: 8.49 ± 1.09 mg/dL Control Group: 10.29 ± 1.41 mg/dL
Pasha et al., 2017 [27]	October2016 - May 2017	Iran	Intervention Group:75 Control Group:75	Intervention Group: Phototherapy + PediLact probiotic (10 drops/day, containing Lactobacillus rhamnosus, Lactobacillus reuteri, Bifidobacterium infantis; administered throughout hospitalization) Control Group: Phototherapy only	Total Bilirubin Levels (mg/dL): Intervention Group:10.25 ± 1.32 mg/dL Control Group: 10.09 ± 1.38 mg/dL (*p* = 0.74)
Ramadan Kamel et al., 2019 [16]	May 2017 - September 2017	Egypt	Intervention Group:50 Control Group:50	Intervention Group: Phototherapy + Lactobacillus probiotic (5 billion CFU/sachet, 3 doses/day for 7 days) Control Group: Phototherapy only	Total Bilirubin Levels (mg/dL): Intervention Group: 4.7 ± 1.4 mg/dL Control Group: 6.3 ± 2.2 mg/dL (*p* < 0.05)
Tariq et al., 2021 [28]	January 2021 - June 2021	Pakistan	Intervention Group:47 Control Group:47	Probiotik + Phototherapy Group: Phototherapy + Half capsule daily of probiotic (Bifidobacterium lactis, Lactobacillus acidophilus, Bifidobacterium bifidum, Lactobacillus rhamnosus) Control Group: Phototherapy only	Total Bilirubin Levels (mg/dL): Intervention Group:16.71 ± 1.25 mg/dL Control Group: 16.76 ± 1.14 mg/dL
Torkaman et al., 2017 [29]	March 2014 - June 2014	Iran	Intervention Group:45 Control Group:47	Intervention Group: Phototherapy + Prokid probiotic (Bifidobacterium lactis, Lactobacillus acidophilus, Bifidobacterium bifidum, Lactobacillus rhamnosus) Control Group: Phototherapy only	Total Bilirubin Levels (mg/dL): Intervention Group:10.10 ± 1.11 mg/dL Control Group: 10.15 ± 1.49 mg/dL (*p* = 0.930)
Tsai et al., 2022 [30]	January 2019 - December 2020	Taiwan	Intervention Group:43 Control Group:40	Intervention Group: Phototherapy + Bifidobacterium animalis subsp. lactis CP-9 (5 × 10⁹ CFU/day, 2 capsules/day with breast milk or formula) Control Group: Phototherapy + Placebo (a capsule containing maltodextrin)	Total Bilirubin Levels (mg/dL): Intervention Group:11.27 ± 0.21 mg/dL Control Group: 11.04 ± 0.19 mg/dL (*p* = 0.417)

### Research ethics

This study is a systematic review and meta-analysis conducted through the systematic examination and quantitative synthesis of previously published scientific research.

### Assessment of methodological quality of the studies

The methodological quality of the studies included in this meta-analysis was analyzed using critical appraisal tools developed by the Joanna Briggs Institute (JBI), tailored to specific research designs. The appraisal tool appropriate to the design of each included study was selected accordingly. For the randomized controlled trials (RCTs) included in this study, the 13-item JBI Critical Appraisal Checklist was employed [31]. Each item was rated using one of four options: “Yes,” “No,” “Unclear,” or “Not Applicable.” The assessment process was independently conducted by two researchers, and any discrepancies in judgment were resolved through discussion and consensus. The quality scores obtained for each study are presented under the heading “Quality Score” in Table 1, and were calculated based on the respective appraisal tool.

### Data synthesis

Comprehensive Meta-Analysis (CMA) Version 2.0 software was used for statistical analysis. Statistical heterogeneity among the included studies was evaluated using the Chi-Square (Q) test and Higgins’ I² statistic. An I² value higher than 50% was considered to indicate significant heterogeneity. The fixed-effects model was applied in cases where I² ≤ 50% and *p* > 0.10, whereas a random-effects model was employed when I² > 50% and *p* > 0.10. Funnel plots were used to examine and visualize publication bias. Egger’s test was also performed to assess publication bias [32, 33].

## Results

All studies included in this meta-analysis employed a randomized controlled experimental design. The total sample size across these studies was 660 for the intervention group and 645 for the control group (Table 1). All included studies met more than 50% of the criteria in the evidence quality assessment tool (Table 1). This finding indicates that the data presented in this study are based on a methodologically sound and reliable research, thereby supporting the scientific validity of the results obtained.

### Meta-analysis results of the studies

Two complementary methods were employed in this meta-analysis to assess the presence of publication bias among the studies.

**Fig. 2:**
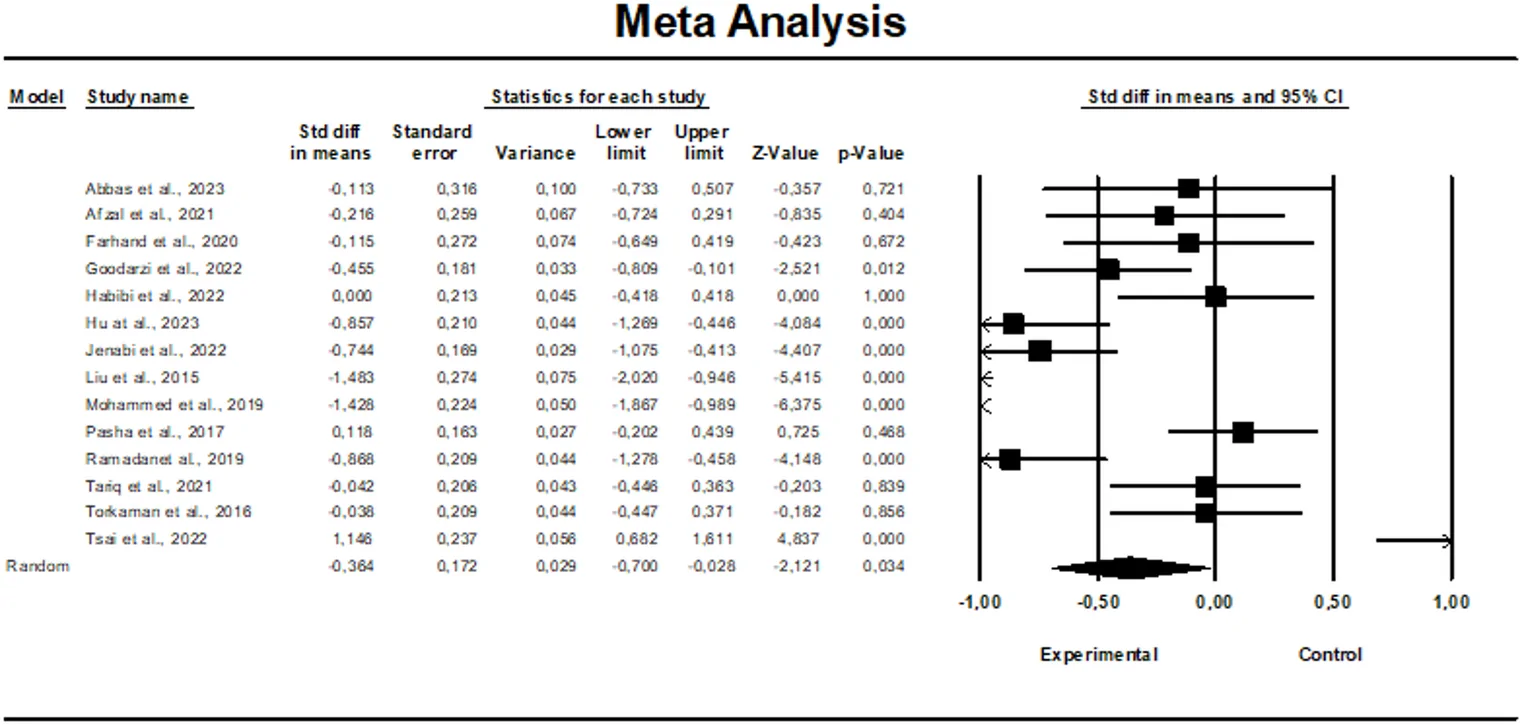
Funnel plot of included studies

First, the distribution of effect sizes was visually examined using a funnel plot (Fig. 2). Then, Egger’s regression test was performed to statistically determine whether publication bias was significant [32]. According to Egger’s method, the intercept (B₀) was − 0.34902 with a 95% confidence interval (CI) ranging between − 10.50277 and 9.80473 (t = 0.07489, degrees of freedom (df) = 12, and the two-tailed p-value = 0.94153). These results indicate that publication bias was not statistically significant.

**Fig. 3:**
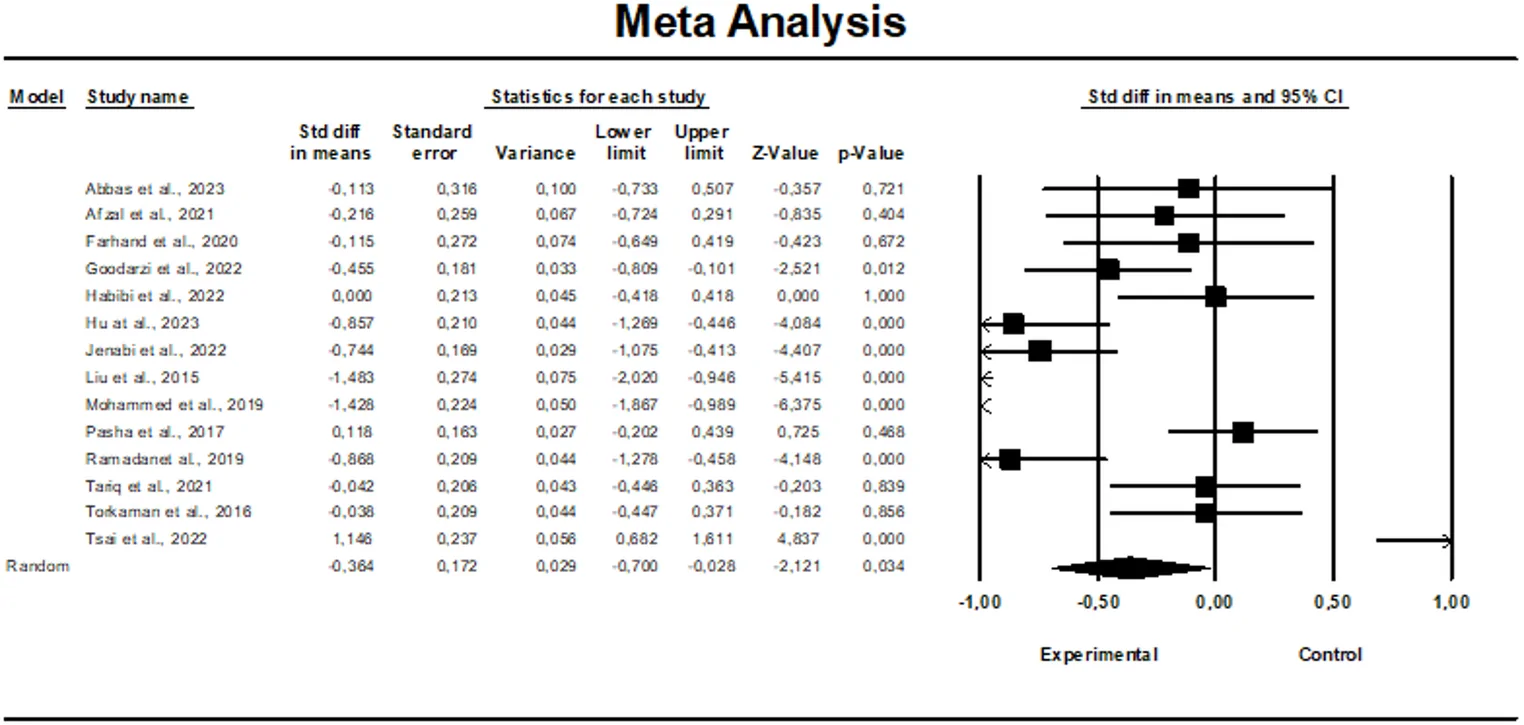
Forest plot of included studies

Figure 3 illustrates the forest plot of 14 studies examining the effect of probiotics on hyperbilirubinemia levels in neonates. The plot includes effect sizes, standard errors, variances, and confidence intervals for each study. The meta-analysis, based on the findings reported by these studies, revealed that probiotics administered in conjunction with phototherapy had a statistically significant effect on reducing hyperbilirubinemia levels in neonates (SMD: -0.364; 95% CI: -0.700 to -0.028; Z = -2.121; *p* = 0.034) (Fig. 3). However, significant heterogeneity was observed among the included studies (I² = 88.678; *p* = 0.000).

## Discussion

This study aims to systematically evaluate evidence on the efficacy of probiotics when used in combination with phototherapy. In this context, 14 studies were examined to determine the effects of probiotic supplementation on neonatal hyperbilirubinemia levels. The meta-analysis results based on these studies revealed that probiotics administered alongside phototherapy significantly reduced hyperbilirubinemia levels in newborns (SMD: -0.364; 95% CI: -0.700 to -0.028; Z = -2.121; *p* = 0.034). However, the very high level of heterogeneity observed across the included studies (I² = 88%) substantially reduces confidence in the pooled estimate and suggests that the magnitude of the effect may vary considerably across different clinical settings. While phototherapy promotes photoisomerization of bilirubin and facilitates its elimination from the body, probiotics may reduce the enterohepatic circulation of bilirubin by modulating the gut microbiota. The synergistic effect of these two approaches may accelerate jaundice treatment, alleviate clinical symptoms in neonates, and play a valuable preventive role. Nevertheless, these potential benefits should be interpreted with caution given the variability in study designs and outcomes. Moreover, this combined therapeutic strategy is considered safe and associated with fewer adverse reactions compared to some conventional treatments [34]. Although probiotics have shown significant potential in managing hyperbilirubinemia, their efficacy remains a topic of ongoing debate [35]. Several studies demonstrated that adding probiotics to phototherapy yields a significant and rapid reduction in both total and indirect bilirubin levels. This effect was particularly observed with strains such as Lactobacillus reuteri and Saccharomyces boulardii [15, 16, 36–38]. However, other studies reported that the reduction in bilirubin levels achieved through probiotic supplementation was not statistically significant or did not yield clinically remarkable benefits [37, 39, 40]. This inconsistency across studies may contribute to the observed heterogeneity and may be explained by differences in probiotic strains, dosages, treatment durations, feeding practices, gestational age, and baseline bilirubin levels.

In a study carried out by Chen et al. in 2017, the efficacy and safety of probiotic supplementation in the treatment of pathological neonatal jaundice were evaluated. As part of this study, 13 randomized controlled trials published up to December 2016 that met predefined inclusion criteria were reviewed, and data from a total of 1,067 neonates were included in the meta-analysis. Clinical outcome variables analyzed in this study included total serum bilirubin levels, duration of jaundice resolution, duration of phototherapy, and length of hospital stay. The findings demonstrated that groups receiving probiotic supplementation exhibited a statistically significant reduction in serum bilirubin levels, as well as notable decreases in the duration of jaundice, phototherapy, and hospitalization. However, the authors noted certain methodological limitations across the included studies, particularly a lack of sufficient clarity regarding randomization and blinding procedures. The results suggest that probiotics may serve as an effective and safe adjunctive approach in the treatment of pathological neonatal jaundice; nonetheless, the authors emphasized the need for longer-term, high-quality, and standardized studies before these findings can be translated into clinical practice [17]. Similarly, the present study also revealed that probiotics significantly reduce hyperbilirubinemia levels in neonates. However, in line with previous meta-analyses, the findings of the present study should be interpreted cautiously due to methodological variability and high heterogeneity.

Another meta-analysis, which completed its literature review in 2017 and was published in 2019, assessed the efficacy and safety of probiotics in reducing the need for phototherapy and the duration of phototherapy in cases of neonatal hyperbilirubinemia. This analysis systematically reviewed randomized controlled trials conducted on neonates of any gestational age and birth weight and included a total of nine studies. The results revealed limited and low-quality evidence regarding the efficacy and safety of probiotic supplementation in infants with neonatal jaundice. Given the findings, the prophylactic use of probiotics did not significantly reduce the incidence of jaundice. Consequently, based on the existing limited and low-quality evidence, the routine use of probiotics for the prevention or treatment of neonatal jaundice is not recommended. The authors stressed the need for additional well-designed and high-quality studies to further evaluate the potential effectiveness of this intervention [11]. Consistent with these findings, the present study supports a potential beneficial effect; however, the strength of this evidence remains limited due to heterogeneity and variability across studies. In this context, the findings of the present study offer significant contributions to the growing body of evidence suggesting that probiotics may be considered as a supportive option in the management of neonatal jaundice. Importantly, these findings should not be interpreted as definitive evidence for routine clinical use.

This literature review also revealed studies investigating the effects of probiotics used alone on hyperbilirubinemia levels. In a similar study, probiotics alone were found to be ineffective in reducing hyperbilirubinemia levels; however, they were reported to promote weight gain in neonates. Since no adverse effects were observed, the use of probiotics was considered safe in neonates [39]. On the other hand, other studies reported that probiotics, even when used alone, can lead to a significant reduction in hyperbilirubinemia levels [41, 42]. Taken together, findings from the literature and the results of the present study indicate that probiotics may be a safe and viable option for reducing hyperbilirubinemia levels in neonates. Nevertheless, further large-scale, well-designed randomized controlled trials with standardized protocols are required to confirm these findings and to better understand the sources of heterogeneity.

### Limitation of studies

This meta-analysis has several methodological and practical limitations. First, the compositions of the probiotic products used in the majority of the included studies were not sufficiently detailed. Specifically, the full names of the bacterial strains, their concentrations, and the extent to which each strain contributed to the observed efficacy were not clearly reported. Moreover, most studies failed to provide information regarding the proportional content of bacterial species present in commercially available probiotic formulations. This lack of detail makes it difficult to identify which specific probiotic strains are more effective in the management of hyperbilirubinemia. In this context, it is necessary for future studies to explicitly report the bacterial species and their concentrations used in interventions. Furthermore, analyzing the individual effects of each strain on phototherapy duration, serum bilirubin levels, and length of hospital stay would offer more precise insights and enhance the clinical applicability of findings.

## Conclusion

The findings achieved in this meta-analysis suggest that probiotic supplementation may represent a potential adjunctive treatment strategy in the management of neonatal hyperbilirubinemia. However, the very high level of heterogeneity observed across studies substantially limits confidence in the pooled estimates and indicates that the magnitude of the effect may vary across different clinical settings. Therefore, these findings should be interpreted with caution. Further well-designed, large-scale randomized controlled trials with standardized protocols are needed to confirm these results and clarify the sources of heterogeneity.

## Data Availability

All data analyzed in this meta-analysis were derived from previously published studies accessible through academic databases. No new datasets were generated.

## Appendix

**Table 2:** Methodological quality assessment of included studies according to JBI criteria

Study	Type of research	Q1	Q2	Q3	Q4	Q5	Q6	Q7	Q8	Q9	Q10	Q11	Q12	Q13	Quality
Abbas et al., 2023 [16]	RCT	✔	NA	✔	NA	✖	✖	✔	✔	✔	✔	✔	✔	✔	9/13
Afzal et al., 2021 [19]	RCT	✔	NA	✔	NA	✖	✖	✔	✔	✔	✔	✔	✔	✔	9/13
Farhand et al., 2020 [20]	RCT	✔	NA	✔	NA	✖	✔	✔	✔	✔	✔	✔	✔	✔	10/13
Goodarz et al., 2022 [21]	RCT	✔	NA	✔	NA	✖	✔	✔	✔	✔	✔	✔	✔	✔	10/13
Habibi et al., 2021 [22]	RCT	✔	NA	✔	NA	✖	✖	✔	✔	✔	✔	✔	✔	✔	9/13
Hu et al., 2023 [23]	RCT	✔	NA	✔	NA	✖	✖	✔	✔	✔	✔	✔	✔	✔	9/13
Jenabi et al., 2022 [24]	RCT	✔	NA	✔	NA	✔	✔	✔	✔	✔	✔	✔	✔	✔	11/13
Liu et al., 2015 [25]	RCT	✔	NA	✔	NA	✖	✖	✔	✔	✔	✔	✔	✔	✔	9/13
Mohammed et al., 2019 [26]	RCT	✔	NA	✔	NA	✖	✖	✔	✔	✔	✔	✔	✔	✔	9/13
Pasha et al., 2017 [27]	RCT	✔	NA	✔	NA	✖	✖	✔	✔	✔	✔	✔	✔	✔	9/13
Ramadan Kamel et al., 2019 [14]	RCT	✔	NA	✔	NA	✖	✖	✔	✔	✔	✔	✔	✔	✔	9/13
Tariq et al., 2021 [28]	RCT	✔	NA	✔	NA	✖	✖	✔	✔	✔	✔	✔	✔	✔	9/13
Torkaman et al., 2017 [29]	RCT	✔	NA	✔	NA	✔	✖	✔	✔	✔	✔	✔	✔	✔	10/13
Abbas et al., 2023 [16]	RCT	✔	NA	✔	NA	✔	✖	✔	✔	✔	✔	✔	✔	✔	10/13

✔: Yes, ✖: No, ?: Unclear, NA: Not Applicable; *Q10–Q13 are only valid for RCTs

## Declarations

### Animal Welfare

Not applicable. This research did not involve the use of animals.

### Clinical trial registration

Not applicable. This study is a meta-analysis and does not involve a clinical trial.

## References

[1] Anderson NB, Calkins KL (2020) Neonatal indirect hyperbilirubinemia. Neoreviews 21(11):e749–e60 10.1542/neo.21-11-e749 33139512

[2] Olusanya BO, Kaplan M, Hansen TW (2018) Neonatal hyperbilirubinaemia: a global perspective. Lancet Child Adolesc Health 2(8):610–620 10.1016/S2352-4642(18)30139-1 30119720

[3] Fanello C, Lee SJ, Bancone G, Kayembe D, Ndjowo P, Badjanga B, Onyamboko M (2023) Prevalence and risk factors of neonatal hyperbilirubinemia in a semi-rural area of the democratic republic of Congo: a cohort study. Am J Trop Med Hyg 109(4):965 10.4269/ajtmh.23-0293 PMC10551084 37669757

[4] Merino-Andrés J, Pérez‐Nombela S, Álvarez‐Bueno C, Hidalgo‐Robles Á, Ruiz‐Becerro I, Fernández‐Rego FJ (2024) Neonatal hyperbilirubinemia and repercussions on neurodevelopment: A systematic review. Child Care Health Dev 50(1):e13183 10.1111/cch.13183 37842871

[5] Zhao H (2017) Multiple organs injury by neonatal hyperbilirubinemia. Wanfang Med Online 44(7):464–466. 10.3760/cma.j.issn.1673-4408.2017.07.006

[6] Bulut O, Erek A, Duruyen S (2022) Effects of hyperbilirubinemia on markers of genotoxicity and total oxidant and antioxidant status in newborns. Drug Chem Toxicol 45(1):451–455 10.1080/01480545.2019.1710182 31902249

[7] Jayanti S, Ghersi-Egea J-F, Strazielle N, Tiribelli C, Gazzin S (2021) Severe neonatal hyperbilirubinemia and the brain: the old but still evolving story. Pediatr Med 4(37):1–20

[8] Zhang M, He Y, Tang J, Dong W, Zhang Y, Zhang B, Mu D (2022) Intensive phototherapy vs. exchange transfusion for the treatment of neonatal hyperbilirubinemia: a multicenter retrospective cohort study. Chin Med J 135(05):598–605 10.1097/CM9.0000000000001962 PMC8920423 35274627

[9] Kliegman RM, St. Geme JW, Blum NJ, Shah SS (2020) Nelson Textbook of Pediatrics. Elsevier, Philadelphia, PA

[10] Akagawa S, Akagawa Y, Yamanouchi S, Teramoto Y, Yasuda M, Fujishiro S, Kaneko K (2021) Association of neonatal jaundice with gut dysbiosis characterized by decreased bifidobacteriales. Metabolites 11(12):887 10.3390/metabo11120887 PMC8705620 34940645

[11] Deshmukh J, Deshmukh M, Patole S (2019) Probiotics for the management of neonatal hyperbilirubinemia: a systematic review of randomized controlled trials. J Maternal-Fetal Neonatal Med 32(1):154–163 10.1080/14767058.2017.1369520 28823200

[12] Zhao X, Mao Y (2022) The Role of Gut Microbiota in the Development and Treatment of Neonatal Hyperbilirubinemia. Front Pead 10:735247

[13] Chen K, Yuan T (2020) The role of microbiota in neonatal hyperbilirubinemia. Am J Translational Res 12(11):7459 PMC7724329 33312382

[14] *Abbas P, Mayangsari CP, Gunawan R (2023) The effect of Lactobacillus acidophilus, Streptococcus thermophilus, and Bifidobacterium longum probiotics on bilirubin levels of neonates with hyperbilirubinemia. Sains Medika: Jurnal Kedokteran dan Kesehatan 14(2):58–65

[15] Ain QU, Mahmood I, Manshah A (2024) Efficacy Of Probiotics In Reducing The Severity And Duration Of Neonatal Hyperbilirubinemia: A Randomized Controlled Trial (RCT). J Bacha Khan Med Coll 5(02):116–121

[16] *Ramadan KT, Abd Ell Atif Afia A, Rifaat Hablas H, Said Al-Shorbagy M (2019) Impact of probiotics on neonatal hyperbilirubinemia. Al-Azhar J Pediatr 22(4):617–628

[17] Chen Z, Zhang L, Zeng L, Yang X, Jiang L, Gui G, Zhang Z (2017) Probiotics supplementation therapy for pathological neonatal jaundice: a systematic review and meta-analysis. Front Pharmacol 8:432 10.3389/fphar.2017.00432 PMC5491971 28713275

[18] Moher D, Liberati A, Tetzlaff J, Altman DG (2009) Preferred reporting items for systematic reviews and meta-analyses: the PRISMA statement. BMJ 339(b2535):1–8 10.1136/bmj.b2535 PMC2714657 19622551

[19] *Afzal T, Butt N, Munir S, Zia N (2021) Effect of addition of probiotics to standard treatment on neonatal jaundice. Ann PIMS-Shaheed Zulfiqar Ali Bhutto Med Univ 17:199–203

[20] *Farhand B, Nayeri ED, Shirzad Kebria T, Ghaffarzadeh Khoei M, Jashni Motlagh A (2020) The effect of Lactoacidophilus probiotic on duration of physiologic jaundice in neonates with 37–42 weeks gestational age: a randomized clinical trial. J Complement Med Res 11:36

[21] *Goodarzi R, Saadat SH, Arshadzadeh M, Darban B, Haghshenas H (2022) Efficacy and safety of probiotics in neonatal hyperbilirubinemia: randomized controlled trial. J Neonatal Nurs 28:286–290

[22] *Habibi M, Sanandaji H, Mojabi SH, Mohammadkhaniha F, Mohammadi N, Mohammadhoseini M (2021) Phototherapy with probiotics supplementation therapy and phototherapy alone in neonates with jaundice: a randomized clinical trial. Immunopathol Persa 8:e2

[23] *Hu D, Wang Y, Yang S, Zhang H (2023) Impact of Saccharomyces boulardii on jaundice in premature infants undergoing phototherapy. J Pediatr (Rio J) 99:263–268 10.1016/j.jped.2022.10.010 PMC10202724 36574954

[24] *Jenabi E, Eghbalian F, Sabzehei MK, Reisi R, Talesh ST (2022) The effect of probiotics and phototherapy combined application in comparison with phototherapy alone on bilirubin reduction in term neonates: a randomized controlled trial (Preprint). Research Square. 10.2103/rs.3.rs-1387582/v1 37929736

[25] *Liu W, Liu H, Wang T, Tang X (2015) Therapeutic effects of probiotics on neonatal jaundice. Pak J Med Sci 31:1172 10.12669/pjms.315.7921 PMC4641277 26649008

[26] *Mohammed Hamed AM, Abdelmeguid MM, Omar SAAM (2019) The effect of probiotics on reducing duration of hospitalization in infants with indirect hyperbilirubinemia. Egypt J Hosp Med 77:5900–5905

[27] *Pasha ZY, Ahmadpour-Kacho M, Ahmadi Jazi A, Gholinia H (2017) Effect of probiotics on serum bilirubin level in term neonates with jaundice: a randomized clinical trial. Int J Pediatr 5:5953–5958

[28] *Tariq B, Shahman M, Mateen A, Kamal MT, Ali AN, Ul Sa S, SHAH H (2021) Role of probiotics in neonates with hyperbilirubinemia receiving phototherapy. Age (days) 6:496

[29] *Torkaman M, Mottaghizadeh F, Khosravi MH, Najafian B, Amirsalari S, Afsharpaiman S (2017) The effect of probiotics on reducing hospitalization duration in infants with hyperbilirubinemia. Iran J Pediatr 27(1):e5096. 10.5812/ijp.5096

[30] *Tsai ML, Lin WY, Chen YT, Lin HY, Ho HH, Kuo YW, Lin HC (2022) Adjuvant probiotic Bifidobacterium animalis subsp. lactis CP-9 improve phototherapeutic treatment outcomes in neonatal jaundice among full-term newborns: a randomized double-blind clinical study. Med (Baltim) 101:e31030 10.1097/MD.0000000000031030 PMC9666203 36397441

[31] Joanna Briggs (2021) Institute critical appraisal tools for use in IBI systematic reviews

[32] Egger M, Smith GD, Schneider M, Minder C (1997) Bias in meta-analysis detected by a simple, graphical test. BMJ 315(7109):629–634 10.1136/bmj.315.7109.629 PMC2127453 9310563

[33] Higgins JP, Thompson SG, Deeks JJ, Altman DG (2003) Measuring inconsistency in meta-analyses. BMJ 327(7414):557–560 10.1136/bmj.327.7414.557 PMC192859 12958120

[34] Li J (2025) Research progress on the application of blue light irradiation combined with oral probiotics in neonatal jaundice. Highlights Sci Eng Technol. 10.54097/6rkp9c47

[35] Milani PG, Nazari A, Soleimani AA, Soleimani RA (2023) Gut–liver axis and the impact of probiotics on neonatal jaundice symptoms: a review. Gastrointest Nurs 21:S10–S15

[36] Khoman DJ, Supriatmo RER, Azlin E (2020) Probiotics role in lowering bilirubin level on hyperbilirubinemia neonates performed phototherapy at neonatology ward of Adam Malik General Hospital. Int J Res Sci Manag 7:59–62

[37] Rezki DA, Firmansyah I, Darussalam D, Yusuf S, Sovira N, Bakhtiar B (2023) The effectiveness of Lactobacillus reuteri on serum bilirubin levels in neonatal hyperbilirubinemia with phototherapy. Paediatr Indones 63:219–225

[38] Suganthi V, Das AG (2016) Role of Saccharomyces boulardii in reduction of neonatal hyperbilirubinemia. J Clin Diagn Res 10:SC12 10.7860/JCDR/2016/20115.8878 PMC5198414 28050461

[39] Santosa I, Shoji H, Itoh S, Shimizu T (2022) Roles of probiotics in reduction of neonatal jaundice in term newborns. Juntendo Med J 68:140–146 10.14789/jmj.JMJ21-0044-OA PMC11189792 38912278

[40] Singh SK, Gupta G, Wasim S, Kumar R, Patra S, Chinmay C (2023) Effectiveness of probiotics and phototherapy compared to phototherapy alone in treating hyperbilirubinemia in neonates ≥ 35 weeks gestation: a comparative observational study. J Neonatol 37:371–377

[41] Bağcı O (2015) Anne sütü sarılığının gelişiminde, anne sütü mikrobiyal içeriği ve bebek barsak mikrobiyal florasının etkisinin araştırılması ve bu bebeklerde probiyotik tedavisinin sarılık seyrine etkisinin değerlendirilmesi. Bursa Uludağ University, Bursa (Turkey)

[42] Irmak E (2015) Probiyotiklerin yenidoğan sarılığı üzerine etkileri. Trabzon (Turkey). Karadeniz Teknik Üniversitesi Tıp Fakültesi

